# Variability in training Experience during orthopaedic surgery post-graduate year-1

**DOI:** 10.15694/mep.2018.0000104.1

**Published:** 2018-05-23

**Authors:** Jeffrey D. Tompson, Jeffrey D. Trojan, Mary K. Mulcahey

**Affiliations:** 1 Department of Orthopaedic Surgery; 2 Department of Orthopaedic Surgery

**Keywords:** Orthopaedic surgery, Surgical skills curriculum, intern year, residency

## Abstract

This article was migrated. The article was marked as recommended.

**Background:** Recently, the orthopaedic surgery PGY-1 curriculum was redesigned to maximize time spent on orthopaedic surgery rotations. Additionally, surgical skills modules were introduced to standardize curricula for orthopaedic interns in the United States.

**Objective:** The purpose of this study is to determine the implementation of the curriculum guidelines were implemented on a national level.

**Methods:** An anonymous survey with 14 multiple choice questions was electronically distributed to program directors (PDs) for all ACGME-accredited orthopaedic surgery residency programs in the US (n=163) in January 2017. Seventy-nine of the 162 PDs (49%) completed the survey in its entirety.

**Results:** The most common non-orthopaedic rotations included: general surgery trauma (67/79=85%), surgical/medical intensive care unit (60/79=76%), plastic and burn surgery (56/79=71%), musculoskeletal radiology (44/79=56%), and vascular surgery (40/79=51%). Seventy-two of 162 residency programs (91%) have a formal surgical skills curriculum for first-year residents, separate from intern boot camp. A variety of training modalities were utilized to teach the interns, most commonly saw bones for fracture fixation (68/79=86%) and cadaveric specimens for surgical approaches (63.79=80%).

**Conclusions:** While all PGY-1 orthopaedic residents are now required to spend six months on orthopaedic rotations, the remaining six months are variable. These non-orthopaedic rotations are likely determined by the PD and available services at the trainee’s institution. This variability has granted PDs the opportunity to individualize the intern experience by maximizing each institution’s assets and designing their own surgical skill curriculum to train their interns using the resources available.

## Introduction

Beginning in July 2013, the American Board of Orthopaedic Surgeons (ABOS) and Accreditation Council for Graduate Medical Education (ACGME) instituted a redesigned PGY-1 curriculum. ^1, 2^ The primary changes introduced more orthopaedic-specific rotations to minimize the time spent on general surgical services. Specifically, the new requirements include: six months of orthopaedic surgery, at least three months of general surgical rotations, and an additional three months of structured training in orthopaedic-related specialties (e.g. anesthesiology, plastic surgery, radiology). Orthopaedic residency programs must also include a surgical skills curriculum that addresses a list of 17 skills and competencies ( Table 1).

While all programs are required to institute these changes to the PGY-1 curriculum to maintain their accreditation, to our knowledge, no studies have examined the differences in implementation on an individual program level. The ACGME and ABOS created guidelines for modification to the intern year curriculum, however, orthopaedic residency program directors (PDs) are allowed to select from a specific set of non-orthopaedic rotations (e.g. general surgery, vascular surgery, emergency medicine) that influence the training and overall quality of the experience for interns prior to commencement of their orthopaedic-specific training in the remaining four years. In addition, a standard surgical skills curriculum has not been defined; rather, orthopaedic residency programs have the opportunity to design a curriculum that meets the needs of their residents.

The purpose of this study was to examine how the ACGME/ABOS guidelines were implemented on a national level. We hypothesized that there would be considerable variability in the non-orthopaedic rotations included in the intern year with regards to the amount of surgical (e.g. general surgery, vascular surgery, plastic surgery, etc.) versus non-surgical (e.g. emergency medicine, internal medicine, physical medicine and rehabilitation, etc.) exposure provided. Additionally, we hypothesized that the structure of the surgical skills curriculum would vary among orthopaedic residency programs.

## Methods

After obtaining IRB approval (Protocol #5152), an anonymous survey was distributed to PDs for all ACGME-accredited orthopaedic surgery residency programs in the United States using SurveyMonkey (San Mateo, CA). At the time of the survey (January 2017), there were 163 orthopaedic residency programs, 155 of which were civilian, and eight of which were military residency programs. Contact information for orthopaedic residency PDs was obtained from publicly-accessible data sources including FREIDA (Fellowship and Residency Electronic Interactive Database), ACGME program listings, and individual program websites. Two follow-up emails were sent, one at two weeks and another at four weeks, to encourage more participation. Participation was entirely voluntary.

The survey contained multiple choice questions designed to gather program demographics including geographic location, university-based versus community-based program, size of each residency class, number of faculty members, and rotation length during the intern year. Additionally, the survey included questions specific to intern year, including identifying all non-orthopaedic rotations, the presence of an intern boot camp at the beginning of the year, and a formal surgical skills curriculum.

### Data Analysis

Data was retrieved and analyzed using the SurveyMonkey web application. Descriptive statistical analysis was performed.

## Results

### Demographics

Electronic surveys were sent to PDs at the 163 ACGME accredited orthopaedic residency programs in the U.S.; however, one survey was returned as undeliverable, leaving 162 programs in the study group. Seventy-nine of 162 PDs (49%) completed the survey in its entirety. Twenty (25%) of the respondents were at programs in the Northeast, 24 (30%) were in the Midwest, 23 (29%) were in the South, and 12 (15%) of the PDs were at residency programs in the West ( Table 2). Many of the responses were from university-based residency programs (n=62, 78%), compared to 16 (20%) from community programs, and one (1%) from a military-based program.

### Program Size

The residency PDs represented programs that varied in size from 0-2 residents per year to classes with more than 11 residents per year ( Table 3). Thirty-six of 79 (46%) programs had 5-7 residents per year, while 28 programs (35%) had 3-4 residents per year. The number of full-time faculty members and the total number of faculty members varied among the programs. Thirteen of 79 programs (17%) had 0-10 full-time faculty, 27 programs (34%) had 11-20 full-time faculty, 22 programs (28%) had 21-30 full-time faculty members, and 17 (22%) had more than 31 full-time faculty members. The total number of faculty members also varied between programs. One program out of 79 (1%) had 0-10 total faculty, 22 programs (28%) had 11-20 total faculty, 27 (34%) had 21-30 total faculty, and 29 (37%) had more than 30 total faculty members.

### PGY-1 Rotations

Sixty-one of 79 programs (77%) had 12 three-to-four week rotations covering the orthopaedic-related specialties, as well as non-orthopaedic surgical and clinical rotations throughout PGY-1. Thirteen programs (17%) had first-year rotations that lasted between five and eight weeks, while five programs (6%) had first-year rotations that were longer than eight weeks. The most common non-orthopaedic rotations included: general surgery trauma (67/79=85%), surgical/medical intensive care unit (60/79=76%), and plastic and burn surgery (56/79=71%) ( Table 4).

### Intern Boot Camp and Surgical Skills Curriculum

Forty-six of 79 programs (58%) held an intern boot camp during the first two months of the academic year, which is separate from clinical rotations. The majority of these programs (27/46=57%) consisted of one-week sessions to train interns in basic surgical and orthopaedic skills.

Seventy-two of 79 residency programs (91%) had a formal surgical skills curriculum for first-year residents, separate from intern boot camp. Nine of the 72 programs (13%) collaborated with other orthopaedic residency programs for the surgical skills curriculum. Forty-one of 72 programs (57%) utilized a year-long curriculum for the surgical skills training, while a dedicated rotation, separate from clinical responsibilities, was used by 31 programs (43%). Twenty-two of the 31 (71%) programs that utilized a dedicated block for surgical skills training had a four-week curriculum, while two-week (5/29=16%), and six-week (2/29=6%) schedules were less common.

Many training modalities were employed to teach first-year residents the surgical skills outlined by the ABOS Surgical Skills Modules including cadavers to practice surgical approaches (63/79=80%) and saw bones to learn the basic skills necessary to perform internal fixation of fractures (68/79=86%) ( Table 5). When teaching basic arthroscopic skills, there is no training modality that was utilized significantly more than any other - virtual simulation (27/79=34%), FAST simulation (35/79=44%), and cadaveric training (32/79=41%).

## Discussion

This study demonstrated substantial variability in the implementation of the ABOS and ACGME curricular requirements in orthopaedic residency programs throughout the United States. A few non-orthopaedic rotations (e.g. general surgery trauma, plastic surgery, radiology, and vascular surgery) were included in the first-year curriculum for over half of the programs; however, the remaining rotations varied considerably between institutions. Roughly half of orthopaedic residency programs offer an intern boot camp during the first two months of PGY-1; however, most of the programs (91%) have a formal surgical skills curriculum for interns, separate from the intern boot camp. In designing surgical skills curricula, most programs include activities to augment didactic and self-directed study such as cadaver labs (80%) and saw bones (86%). There is wide variability in the use of arthroscopic simulation (e.g. virtual reality, FAST workstation, and cadavers), with programs likely utilizing more than one modality.

Since 2013, the ABOS and ACGME have implemented major changes to the curricular requirements for first-year orthopaedic residents, including revisions to the PGY-1 rotation schedule. ^1- 3^ By increasing the orthopaedic surgery rotations from three- to six-months, first-year residents spend more time working directly with upper-level residents and attendings, providing the interns with increased exposure to both inpatient and outpatient clinics, as well as the operating room. In addition to changes to the rotation schedule, the 2013 modifications required programs to create basic surgical skills curricula designed around 17 modules at the discretion of the PD, utilizing the resources available to each residency program.

Numerous publications describe the initial experience with the design and implementation of a surgical skills curriculum into orthopaedic residency programs. Karam et al. detailed the experience at the University of Iowa, which created one of the first month-long surgical skills training programs for PGY-1 residents. ^4^ The authors concluded that both residents and faculty members found the initial iteration of the surgical skills curriculum to be an effective teaching model that could be improved with minor refinements from year to year. Ford et al. described the surgical skills curriculum designed to train orthopaedic interns at Carolinas Medical Center. ^5^ The study described numerous details about the surgical training program including total cost ($8,100) and time spent by the residents practicing the surgical skills (89 hours). Overall, the residents were satisfied with the structure of the curriculum, with the average module receiving 4.15 points out of 5 (4 represented “good” and 5 represented “excellent”). The authors also described the invaluable benefit of early intern-attending interactions, allowing for the development of clinical and research relationships during the first year of residency. A third study conducted at the University of Toronto demonstrated similar beneficial results of an intern boot camp after dividing the first-year residents into three groups - on-service, off-service, and intensive skills laboratory groups. ^6^ The residents in the skills laboratory group performed significantly better than both residents on and off orthopaedic services. While the implementation of surgical skills curricula seems to be well-received by orthopaedic residents and faculty, the study was unable to quantify the effect, thereby stating that it is unclear if these programs lead to an overall improvement in surgical skills and a transfer of techniques learned in the laboratory to the operating room. In 2015, a study by Jones et al. compared scores in task-specific measures between PGY-1 residents following a three-month orthopaedic “intern boot camp” and PGY-2 residents immediately following their orthopaedic internship that did not include the surgical skills curriculum. ^7^ The authors found that there was no difference in performance during the surgical skill examinations between the two groups.

In addition to examining the effect of an overarching surgical skills curriculum, there have been studies that examine the ability of cadaveric instruction and surgical simulators to aid in the instruction of surgical residents. A study by Van Heest et al. in 2009 evaluated the ability of orthopaedic surgery residents to complete a cadaveric carpal tunnel release. The authors found a significant difference between year of training and ability to adhere to a detailed surgical checklist. ^8^ A separate study demonstrated the improvement of diagnostic shoulder arthroscopy skills and competency after residents had completed Virtual Reality (VR) simulated training sessions. ^9^ Another recent study detailed the current state of simulation training in orthopaedics and concluded that simulation is an effective training modality to improve technical skills. Furthermore, this study described a push to include an independent surgical skill evaluation during the orthopaedic surgery board certification process. ^10^

There are several limitations to this study. First, only seventy-nine of the 162 PDs (49%) responded, therefore, these results may not be generalizable to all of the orthopaedic residency programs in the United States. However, several previously published studies that surveyed orthopaedic residency PDs demonstrate similar response rates, varying from 16-56%. ^11- 13^ Second, this survey study is subject to the limitations that are inherent to this type of investigation, including failure to complete the survey by some respondents and varied interpretations of the questions. To address this limitation, the survey was designed such that all questions required a response, thereby preventing the respondent from skipping any questions. Additionally, all survey questions included multiple choice responses, thereby limiting the amount of individual interpretation by PDs.

## Conclusion

While all PGY-1 orthopaedic residents are now required to spend six months on orthopaedic rotations, the remaining six months of the intern schedule consist of non-orthopaedic rotations that are determined both by the residency PD and, likely, the services available at the trainee’s institution. This variability has granted PDs the opportunity to individualize the intern experience to maximize the assets of the given institution. Additionally, although the skills deemed necessary for a PGY-1 orthopaedic resident have been defined by the ABOS surgical skills modules, each residency program can design their own curriculum to train their interns using the resources available.

## Notes On Contributors

Jeffrey D. Tompson is an orthopaedic surgery resident at Rutgers New Jersey Medical School.

Jeffrey D. Trojan is a research assistant in the Department of Orthopaedic Surgery at Tulane University School of Medicine.

Mary K. Mulcahey is an associate professor and the director of the Women’s Sports Medicine Program in the Department of Orthopaedic Surgery at Tulane University School of Medicine.

## Appendices

**Table 1.** Modules for the ABOS Surgical Skills Curriculum.

Module	Content
1	Sterile technique - operating room setup
2	Suturing and knot tying
3	Microsurgical suturing techniques
4	Soft tissue handling and dissection
5	Casting and splinting: splints, casts, and removal
6	Traction techniques
7	Compartment syndrome: diagnosis and treatment
8	Bone handling techniques - osteotomy
9	Fluoroscopic knowledge and skills
10	K-Wire techniques
11	Techniques basic to internal fixation of fractures
12	Principles and techniques of fracture reduction
13	Basic techniques in external fixation
14	Basic arthroscopy skills
15	Basic arthroplasty skills (TKA and THA)
16	Joint aspiration and injection
17	Patient safety, team training, and obtaining consent

**Table 2.** Geographic distribution of program directors responding to the survey.

US Census Region	Responses	Percentage
Northeast - New England (CT, ME, MA, NH, RI, VT)	7	9%
Northeast - Middle Atlantic (NJ, NY, PA)	13	17%
Midwest - East North Central (IN, IL, MI, OH, WI)	18	23%
Midwest - West North Central (IA, KS, MN, MO, NE, ND, SD)	6	8%
South - South Atlantic (DE, DC, FL, GA, MD, NC, SC, VA, WV)	13	17%
South - East South Central (AL, KY, MS, TN)	2	3%
South - West South Central (AR, LA, OK, TX)	8	10%
West - Mountain (AZ, CO, ID, NM, MT, UT, NV, WY)	5	6%
West - Pacific (AK, CA, HI, OR, WA)	7	9%

**Table 3.** Distribution of class size for orthopaedic residency programs.

Number of Residents	Responses	Percentage
0-2	8	10%
3-4	28	35%
5-7	36	46%
8-10	6	8%
11+	1	1%

**Table 4.** Non-orthopaedic rotations completed during the intern year.

Non-Orthopaedic Rotation	Response	Percentage
Anesthesiology	34	43%
Basic Surgical Skills	29	37%
Emergency Medicine	36	46%
General Surgery	39	49%
General Surgery Trauma	67	85%
Internal Medicine	8	10%
Introduction to Research	1	1%
Musculoskeletal Radiology	44	56%
Neurological Surgery	26	33%
Pediatric Surgery	15	19%
Physical Medicine and Rehabilitation	16	20%
Plastic/Burn Surgery	56	71%
Surgical/Medical ICU	60	76%
Rheumatology	9	11%
Vascular Surgery	40	51%

**Table 5.** Training modalities utilized to assist in teaching skills highlighted in the ABOS Surgical Skills Curriculum.

Training Modality	Response	Percentage
Arthroscopic simulation - virtual reality	27	34%
Arthroscopic simulation - FAST workstation	35	44%
Arthroscopic simulation - cadaver	32	41%
Cadavers - surgical approaches	63	80%
Cadavers - implantation of hardware	45	57%
Informed consent	34	43%
Saw bones	68	86%
Standardized patients	13	17%

## References

[1] ABOS surgical skills modules for PGY-1 residents . American Board of Orthopaedic Surgeons. Available at: https://www.abos.org/residency-programs/residency-skills-modules.aspx Accessed: February 2017.

[2] Accreditation Council for Graduate Medical Education . ACGME program requirements for graduate medical education in orthopaedic surgery. 2016:1–30.

[3] Dougherty PJ Marcus RE . ACGME and ABOS changes for the orthopaedic surgery PGY-1 (intern) year. Clin Orthop Relat Res. 2013;471(11):3412–6. 10.1007/s11999-013-3227-9 23934034 PMC3792253

[4] Karam MD Westerlund B Anderson DD Marsh JL . Development of an orthopaedic surgical skills curriculum for post-graduate year one resident learners - the University of Iowa experience. Iowa Orthop J. 2013;33:178–84. 24027480 PMC3748876

[5] Ford SE Patt JC Scannell BP . A comprehensive, high-quality orthopedic intern surgical skills program. J Surg Ed. 2016;73(4):553–8. 10.1016/j.jsurg.2016.03.008 27142722

[6] Sonnadara RR Van Vilet A Safir O Alman B Ferguson P Kraemer W Reznick R . Orthopedic boot camp: examining the effectiveness of an intensive surgical skills course. Surgery. 2011;149(6);745–9. 10.1016/j.surg.2010.11.011 21236456

[7] Seeley MA Kazarian E King B Biermann JS Caird MS Irwin TA . Core concepts: orthopedic intern curriculum bootcamp. Orthopedics. 2016;39(1):e62–7. 10.3928/01477447-20151228-03 26730688

[8] Van Heest A Putnam M Agel J Shanedling J McPherson S Schmitz C . Assessment of technical skills in orthopaedic surgery residents performing open carpal tunnel release surgery. J Bone Joint Surg Am. 2009 Dec;91(12):2911–7. 10.2106/JBJS.I.00024 19952242

[9] Waterman BR Martin KD Cameron KL Owens BD Belmont PJ . Simulation training improves surgical proficiency and safety during diagnostic shoulder arthroscopy performed by residents. Orthopedics. 2016 May;39(3):e479–85. 10.3928/01477447-20160427-02 27135460

[10] Kivanc A Satava RM Van Heest A Hogan MV Pedowitz RA Fu F Sitnikov I Marsh JL Hurwitz SR . Retention of skills after simulation-based training in orthopaedic surgery. J Am Acad Orthop Surg. 2016 Aug;24(8):505–14. 10.5435/JAAOS-D-15-00440 27348146

[11] Hall MP Kaplan KM Gorczynski CT Zuckerman JD Rosen JE . Assessment of arthroscopic training in U.S. orthopedic surgery residency programs - a resident self-assessment. Bull NYU Hosp Jt Dis. 2010;68(1):5–10. 20345354

[12] Immerman I Kubiak EN Zuckerman JD . Resident work-hour rules: a survey of residents’ and program directors’ opinions and attitudes. Am J Orthop (Belle Mead NJ). 2007;36(12):e172–9. 18264560

[13] Karam MD Pedowitz RA Natividad H Murray J Marsh JL . Current and future use of surgical skills training laboratories in orthopaedic resident education: a national survey. J Bone Joint Surg Am. 2013;95(1):e4( 1-8). 23283381 10.2106/JBJS.L.00177

