# Supplementary material for: Variability in training Experience during orthopaedic surgery post-graduate year-1
Source: MedEdPublish (2016). 2018 May 23;7:104. [Version 1] doi: 10.15694/mep.2018.0000104.1 (PMC10699375; doi:10.15694/mep.2018.0000104.1)
Supplement: Peer review history

# Reviewer report: Reviewer response for version 1

**DOI:** 10.21956/mep.19578.r29055

**Published:** 2019-05-08

**Authors:** Trevor Gibbs

**Affiliations:** 1 AMEE

This review has been migrated. The reviewer awarded 4 stars out of 5 An interesting and well written paper. As we move more and more to super-specialities, the "add-ons" that such such subjects as orthopeadic surgery become important. I imagine that frequently it is a difficult choice for programe directors and there becomes a balance between standardisation and interesting others. I think this is an important paper since it brings to the fore this discussion need. Personally I feel that there are surgical subjects that are closer to orthopeadic sugery than others and it should be these that control what other surgical subjects are included

Reviewer Expertise:

NA

No decision status is available

# Reviewer report: Reviewer response for version 1

**DOI:** 10.21956/mep.19578.r29054

**Published:** 2018-06-28

**Authors:** David Bruce

**Affiliations:** 1 None

This review has been migrated. The reviewer awarded 3 stars out of 5 This paper will be of interest for those involved in training for orthopaedics and organisers of training programmes where a change on the programme has been mandated by their specialty and accrediting organisations. The survey demonstrates that within the parameters set in terms of learning experiences and outcomes, there is considerable flexibility in the organisation of of the training programmes. While this gives a useful baseline measurement, it would have been interesting to know how each training provider developed their new programmes. It is not clear whether these are based on availability of rotations and issues such as faculty for surgical skills boot camps, or if not, what criteria was used to design the programmes. The paper is well written and the demonstrated variation in organisation across the training programmes would be a good baseline for a further study to consider the experiences of the trainees in these differing programmes.

Reviewer Expertise:

NA

No decision status is available
